# Supplementary material for: Modeling the spread of fake news on Twitter
Source: PLoS One. 2021 Apr 22;16(4):e0250419. doi: 10.1371/journal.pone.0250419 (PMC8062041; doi:10.1371/journal.pone.0250419)
Supplement: S1 Appendix — We summarize the baseline methods for predicting the evolution of the spread of a fake news item: linear regression (LR) and reinforced Poisson process (RPP). (PDF) [file pone.0250419.s001.pdf]

S1 Appendix: Baseline methods We summarize the baseline methods for predicting the evolution of the spread of a fake news item: linear regression (LR) and reinforced Poisson process (RPP).

Linear regression (LR)

Linear regression is applied to the logarithm of the cumulative number of posts up to time t:

$$\log R_t = \alpha_t + \log R(T_{\mathrm{obs}}) + \sigma_t \xi_t,$$

where R_t is the cumulative number of posts at the prediction time t, $R(T_{\mathrm{obs}})$ is the cumulative number of posts at the observation time T_{obs}, and ξ_t represents the Gaussian random variable with zero mean and unit variance. The parameters $\{\alpha_t, \sigma_t^2\}$

are estimated by the maximum likelihood method from the training data where the tweet sequence in the entire period is available. The cumulative number of posts is predicted by the unbiased estimator

$$\hat{R}_t = R(T_{\mathrm{obs}}) \exp(\hat{\alpha}_t + \hat{\sigma}_t^2/2),$$

where $\hat{R}_t$ is the prediction of the cumulative number, and $\hat{\alpha}_t$ and $\hat{\sigma}_t^2$ are the fitted parameters.

Reinforced Poisson process (RPP)

RPP is a point process model, similar to TiDeH, where the instantaneous function is written as

$$\lambda(t) = c f_\gamma(t) r_\alpha(R(t)),$$

where $f_\gamma(t) = t^{-\gamma}$ describes the aging effect, and $r_\alpha(R) = \epsilon + \frac{1-e^{-\alpha(R+1)}}{1-e^{-\alpha}}$ is a reinforcement mechanism associated with the multiplicative nature of the spreading. The model parameters $\{c, \gamma, \alpha\}$ are determined by the maximum likelihood method. The cumulative number of posts is evaluated by the expectation of the RPP model, described as follows:

$$\frac{dR}{dt} = \lambda(t)$$

which can be solved analytically

$$R(t) = (\log(1 + e^x) - x - \log \tilde{\epsilon} - \alpha)/\alpha,$$

with

$$x(t) = \frac{\tilde{\epsilon} c \alpha (T_{\mathrm{obs}}^{1-\gamma} - t^{1-\gamma})}{(1-\gamma)(1-e^{-\alpha})} - (R(T_{\mathrm{obs}}) + 1)\alpha - \log(\tilde{\epsilon} - e^{-\alpha(R(T_{\mathrm{obs}})+1)}),$$

and $\tilde{\epsilon} = 1 + \epsilon(1 - e^{-\alpha})$. This expression is used to predict the cumulative number.
